# The Effectiveness of Semi-Automated and Fully Automatic Segmentation for Inferior Alveolar Canal Localization on CBCT Scans: A Systematic Review

**DOI:** 10.3390/ijerph19010560

**Published:** 2022-01-04

**Authors:** Julien Issa, Raphael Olszewski, Marta Dyszkiewicz-Konwińska

**Affiliations:** 1 Department of Biomaterials and Experimental Dentistry, Poznań University of Medical Sciences, Bukowska 70, 60-812 Poznań, Poland; m.dyszkiewicz@ump.edu.pl; 2 Department of Oral and Maxilofacial Surgery, Cliniques Universitaires Saint Luc, UCLouvain, Av. Hippocrate 10, 1200 Brussels, Belgium; raphael.olszewski@uclouvain.be; 3 Oral and Maxillofacial Surgery Research Lab (OMFS Lab), NMSK, Institut de Recherche Experimentale et Clinique, UCLouvain, Louvain-la-Neuve, 1348 Brussels, Belgium

**Keywords:** artificial intelligence, algorithm, inferior alveolar nerve, CBCT

## Abstract

This systematic review aims to identify the available semi-automatic and fully automatic algorithms for inferior alveolar canal localization as well as to present their diagnostic accuracy. Articles related to inferior alveolar nerve/canal localization using methods based on artificial intelligence (semi-automated and fully automated) were collected electronically from five different databases (PubMed, Medline, Web of Science, Cochrane, and Scopus). Two independent reviewers screened the titles and abstracts of the collected data, stored in EndnoteX7, against the inclusion criteria. Afterward, the included articles have been critically appraised to assess the quality of the studies using the Quality Assessment of Diagnostic Accuracy Studies-2 (QUADAS-2) tool. Seven studies were included following the deduplication and screening against exclusion criteria of the 990 initially collected articles. In total, 1288 human cone-beam computed tomography (CBCT) scans were investigated for inferior alveolar canal localization using different algorithms and compared to the results obtained from manual tracing executed by experts in the field. The reported values for diagnostic accuracy of the used algorithms were extracted. A wide range of testing measures was implemented in the analyzed studies, while some of the expected indexes were still missing in the results. Future studies should consider the new artificial intelligence guidelines to ensure proper methodology, reporting, results, and validation.

## 1. Introduction

Artificial intelligence (AI) is a broad domain combining the science and engineering of developing intelligent systems and machines [1,2] that can accomplish complex human cognitive functions such as problem-solving, structure and word recognition, and decision making [3]. The AI has become integrated into our daily life directly and indirectly through digital assistance (Apple’s Siri, Google Now, Amazon’s Alexa, Microsoft’s Cortana…), online recommendations (music, products, movies, map navigation, etc.), advertisements, email filtering, smart replies, automatic detection and other essential fields such as medicine where it is in continuous development [4,5,6]. Machine learning, a subdivision of AI, enables algorithms to learn and predict from data patterns, whereas deep learning enables this process using larger raw data [7,8]. 

In order to make the most accurate knowledge-based decision, higher experience and data analysis are required [9]. Based on this concept, AI is being implemented extensively in medicine, particularly in diagnosis and decision-making [8,9]. Two forms of AI exist in the medical field: virtual (electronic health records, diagnostic and treatment planning software, and others) and physical (robot surgery assistance, smart prostheses, etc.) [1,10]. Moreover, AI applications in dentistry are rapidly growing [11]. They are used for caries detection and diagnosis [12], oral cancer screening [13,14], improvement of brushing method [15], management of dental fear [16], automatic cleaning, shaping, and filling of the root canal [17], differential diagnosis, treatment planning, and detection of anatomical structure on dental radiographic data [18].

The knowledge of dentists about the basics of dental tomography and the use of cone-beam computed tomography (CBCT) remains questionable despite its popularity in dentistry [19] due to the lack of uniformity of the dental curriculum across dental schools worldwide. Particularly, the exclusion of the CBCT topic from undergraduate studies in some countries and the lack of specialists from the oral and maxillofacial radiology in most European countries [19] raised the question of whether, despite the growing number of CBCT machines, dentists are prepared for the diagnostic process [20]. In consequence, dentists seek additional training and are also becoming interested in available tools that could assist them in the process of reporting. Researchers proposed the use of artificial intelligence (AI) as a fast-assisting tool for dentists in reading and reporting two-dimensional (2D) and three-dimensional (3D) radiographic scans [21,22].

The inferior alveolar nerve (IAN) is an essential nerve that resides in the mandibular canal (MC), which is also known as the inferior alveolar canal (IAC), along with the artery and veins [23]. The IAN, as well as the MC, exhibits different path variations [24,25]. In order to avoid any IAN injuries that may vary from temporary nerve numbness with or without paresthesia to permanent nerve paresthesia (with or without trigeminal neuralgia) [26], a proper tracing on the radiographic image could be helpful [27]. In particular, using CBCT that delivers 3D images [28] gives the operator a choice to evaluate the scanned structures from different views, allowing proper assessment of the IAC and tracing of IAN [29].

Hung et al. [30], in their review investigating the clinical applications and diagnostic performance of AI in dental and maxillofacial radiology, emphasized the need for future systematic reviews describing and assessing the value, impact, and reliability of AI in daily practice. Furthermore, as the implementation of AI in dentistry is relatively new, it is essential to investigate its ability to detect or predict disease or confirm physiological presentation, to increase diagnostic test accuracy, and to compare it to a gold standard test [31]. In this review, we aim to present and systematically analyze the effectiveness of semi-automatic and fully automatic methods for IAN/IAC localization together with future recommendations for practitioners and researchers.

## 2. Materials and Methods

The proposed systematic review is conducted in accordance with Joanna Briggs Institute (JBI) methodology [32] for diagnostic test accuracy as well as in accordance with PRISMA (Preferred Reporting Items for Systematic Reviews and Meta-Analyses) guidelines [33]. The objective of the review is to identify the available semi-automatic and fully automatic algorithms for IAC localization as well as to present their diagnostic accuracy. The component of the mnemonic PIRD [34] (Population, Index test, Reference test, and Diagnosis of interest) were established as follows:

- Population: CBCT scans of oral and maxillofacial area in humans.
- Index test: Diagnostic tool based on semi-automatic and fully automatic algorithm.
- Reference test: Experts judge or manual tracing.
- Diagnosis of Interest: IAC/IAN localization.

### 2.1. Searching Strategy

Five different databases (PubMed, Medline, Web of Science, Cochrane, and Scopus) have been searched electronically until the 14 using a complete searching strategy (Table S1). The implemented searching strategy has been developed and customized for each database after a limited primary search, including the following MeSH keywords: “algorithm” OR” algorithm*” OR “artificial intelligence” OR “AI” OR “automatic” OR “automated” OR “semi-automatic” OR “semi-automated” OR “deep learning” OR “Convolutional neural network” OR CNN OR “machine learning” AND “mandibular canal” OR “inferior alveolar canal” OR “inferior alveolar nerve.” All the retrieved articles were imported to EndNote X7 (Clarivate Analytics, PA, USA) library, and library de-duplication was applied according to Bramer et al. [35].

### 2.2. Eligibility Criteria

The inclusion and exclusion criteria have been based on the mnemonic PIRD [32,34]. The retrospective clinical trials, cross-sectional and case-control studies investigating the accuracy of diagnostic tools based on semi-automatic or fully automatic algorithms on human CBCT scans for tracing the IAN and comparing it to manual techniques performed by the expert judges were included. In contrast, the exclusion criteria include pilot studies, ex-vivo studies, and conference papers. Additionally, studies investigating orthopantomography or computed tomography (CT) scans as well as studies on animals were excluded. (Table 1).

As the review question is considered innovative and new in the field, no date or language restrictions have been used.

### 2.3. Study Selection

Two independent reviewers (J.I and M.D.K) screened the title and abstract of the collected data against the inclusive criteria after a pilot test of the method. The potential articles resulting from the primary screening have been kept, and the full text was assessed in detail according to the inclusive criteria by the same reviewers independently. Any disagreements that arise between the two reviewers at any stage of the process were resolved through discussion or with the third reviewer (R.O).

### 2.4. Critical Appraisal and Data Extraction

Based on the JBI recommendation [32] and Ma et al. review [36], the QUADAS-2 (Quality Assessment of Diagnostic Accuracy Studies-2) (Table S2) tool has been used to exam the methodology of the included studies against the predefined criterion, with the aim of considering individual sources of risk of bias. The QUADAS-2 question has been answered by ‘Yes’, ‘No’, ‘Unclear’, or, on some occasions, ‘Not applicable’. Before the appraisal process, the reviewers have agreed on specific criteria to be implemented for the inclusion or exclusion of any study from the review; this criterion was then applied consistently across studies.

The data extraction was performed by one reviewer (J.I) and evaluated independently by the second reviewer (M.D.K). The extracted data are presented in Table 2. It includes the author(s), year of publication, study location, study methodology, sample size, persons executing and interpreting index tests (numbers, training, and expertise), as well as the following reported values, were extracted according to availability sensitivity, specificity, accuracy, and the agreement level between both methods. 

## 3. Results

### 3.1. Search Result

**Figure 1:**
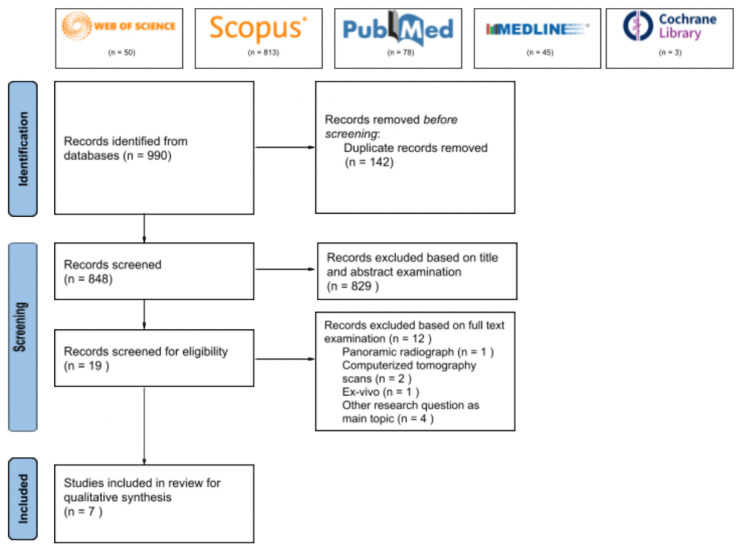
PRISMA flow diagram for the systematic reviews, which included searches of databases.

A total of 990 articles were collected on the 22 of August 2021 from five electronic databases (PubMed, Medline, Web of Science, Cochrane, and Scopus). After the removal of 142 article duplicates, the title and abstract of 848 articles were evaluated against the inclusion and exclusion criteria resulting in 19 articles eligible for full-text assessment. Following full-text evaluations, only seven pieces have been qualified for the systematic review and subjected to final screening using the QUADAS-2 instrument (Figure 1). The inter-reviewer reliability, kappa statistics, K = 0.883 indicate a significant agreement between the reviewers. 

All seven retrospective studies involve a total of 1288 human CBCT scans. Five out of seven studies used convolutional neural network algorithms [37,38,39,40,41], and in the other two studies, one used statistical shape models [42], and the other one tested a new automated method [43]. Despite the progress of AI within oral and maxillofacial radiology, the number of published studies testing AI algorithms for IAN/IANC detection on CBCT scans is relevantly low; from 2016 till the 22 of August 2021, only seven studies have been published and identified. 

The U-net-like algorithms implemented by Diagnocat software (Diagnocat Inc, West Sacramento, CA, USA) were tested by Orhan et al. [37] and Bayrakdar et al. [39], respectively tested 85 and 75 CBCT scans as sample size. In each study, one oral and maxillofacial radiologist was involved in performing the reference test.

Using a total sample size of 637 CBCT scans divided as follows 457 scans for the training set, 52 scans validation set, and 128 CBCT scant as test set, Jaskari et al. [41] tested the fully convolutional deep neural network algorithm. The reference test was carried by one dental and maxillofacial radiologist with 34 years of experience and a resident in oral and maxillofacial radiologist with ten years of experience using Romexis^®^ 4.6.2.R software (Planmeca, Helsinki, Finland) for IAN annotation.

Liu et al. [38] used two U-Nets and One ResNet-34 in their proposed approach, consisting of two modules, one for MC and third molar detection while the other for MC and third molar relation classification. The total sample size included a total of 229 CBCT scans divided into 154 scans for training, 30 scans for validation, and the rest 45 scans for testing. Two oral and maxillofacial radiologists with ten years of experience performed the reference test, the modification of the primary segmentation was completed manually using Multi-Planar Reformation (MPR). 

Kwak et al. [40] tested three different algorithms, 2D SegNet, 2D U-Net, and 3D U-Net, using a total of 102 CBCT scans of patients ranging from 18 to 90 years old. The sample size was split into three sets in the following ratios 6:2:2 (training set: validation set: testing set). The reference test in this study has been performed by two trained researchers and one oral and maxillofacial radiology with six years of experience using INVIVO™ (Anatomage, San Jose, CA, USA).

Statistical shape models were tested by Abdolali et al. [42], the sample consisted of 120 CBCT scans, and two radiologists were conducting the reference test. 

Bahrampour et al. [43] proposed a new automated algorithm and tested it using a sample of 40 CBCT scans. Two maxillofacial radiologists performed the reference test. 

The number of experts involved in tracing the IAC varied from 1 to 3 evaluators ranging from radiologists, oral maxillofacial radiologists, and residents in oral maxillofacial radiology. The reference test results were then compared to the results of the tested algorithms. The sensitivity (90.2%) and specificity (95%) were only reported in Lui et al. [38] study, while three studies [38,40,41] reported the accuracy without presenting the diagnostic odds. Kappa statistics and Kendall’s coefficient were reported respectively by Orhan et al. [37] (0.762) and Liu et al. [38] (0.901) in their studies to describe the level of agreement between the index and reference test. Liu et al. [38] determined the reliability between the two investigators using Weighted Kappa (0.783) that indicated good results. The extracted data from the studies are described in Table 2.

### 3.2. Risk of Bias

**Figure 2:**
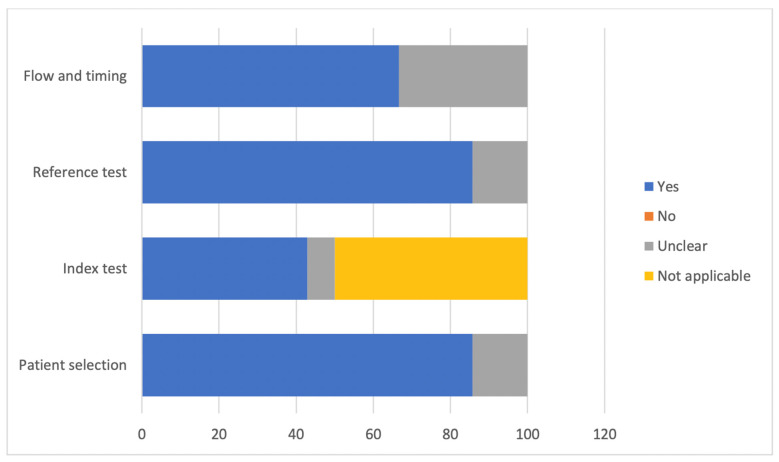
Risk of bias.

Based on the Quality Assessment of Diagnostic Accuracy Studies-2 (QUADAS-2) tool, all studies demonstrated a low to moderate risk of bias. The detailed quality assessment is shown in Figure 2.

## 4. Discussion

The major weaknesses for most of the selected and analyzed studies were the variation of indexes used for result presentation [37,38,39,40,41,42,43], the absence of clear exclusion criteria [37,38,39,42,43], and poor explanation of the reference test [37,39,42,43]. These weaknesses mainly affect the studies’ duplication process that is essential according to the standards for reporting of diagnostic accuracy studies (STARD) guidelines [44].

The used samples were from the same setting or location [37,39,40], and the accuracy of the training sets haven’t been described extensively [37,39,43]. It is worth noting that accurate results are expected with more extensive training sets because insufficient sample for training may lead to over-fitting and reducing the ability of the algorithm in generalizing unseen data [45]. The inter-observer reliability was only reported in Liu et al. [38] study, using weighted kappa (k = 0.783). It should be emphasized that reporting the inter-rater and the intra-rater reliability would be beneficial to assess the reproducibility of each observer and the overall agreement between observers [46,47].

Analyzing the design, the methodology, and reported results of the seven studies [37,38,39,40,41,42,43], we have noted that the authors did not follow any defined guidelines. The reported accuracy of the diagnostic test in three studies [38,40,41] was given without presenting the diagnostic odds. In contrast, diagnostic values (true positive, false negative, true negative, false positive) are mandatory to ensure a complete evaluation of the test accuracy [48].

Considering the frequent CBCT artifacts (noise, extinction artifacts, beam hardening, scattering, motion artifacts, etc.) and their impact on diagnosing [49], testing the accuracy of the algorithm on a set of CBCT scans including these artifacts is essential for future clinical application. In our review, none of the included studies considered this category in their samples, while Liu et al. [38] excluded blurred CBCT images caused by artifacts. 

The principal research guidelines didn’t include the AI section as they had been established before the development of AI. This justifies the high frequency of unclear and not applicable answers in our review, to the QUADAS-2 tool questions. For example, the index test section gave 50% of not applicable and 7.14% of unclear answers as the QUADAS-2 tool wasn’t designed to evaluate the risk of bias for AI diagnostic accuracy studies [50].

The number of studies testing the accuracy of the AI in dentistry, especially in oral and maxillofacial radiology, is increasing alongside the addition of the AI sections within the research guidelines. Recently, Sounderajah et al. [51] started developing AI-specific extensions for STARD guidelines, EQUATOR (Enhancing Quality and Transparency of Health Research), and TRIPOD (Transparent Reporting of a Multivariable Prediction Model for Individual Prognosis or Diagnosis). Furthermore, the AI extension for SPIRIT (Standard Protocol Items: Recommendations for Interventional Trials) [52] and CONSORT (Consolidated Standards of Reporting Trials) [53] have been developed, published, and need to be endorsed by journals aiming to improve the quality of dental AI research [54]. A recent checklist by Schwendicke et al. [55], has been published in order to guide researchers, reviewers, and readers.

## 5. Conclusions

In summary, we encourage researchers to consider the limitations mentioned above as they may lead to bias in evaluating the used algorithm power and to follow the AI guidelines that are consistently updated. Especially in the view of the benefits from implementing AI, which could allow a global uniformity of the dental report and would assist dentists in their efforts, saving their time but keeping the quality for better outcomes. This review could be viewed as a preliminary report to guide researchers while investigating AI in order to obtain accurate results allowing the proper evaluation of the given algorithm. 

## Figures and Tables

**Table 1:** Table of Inclusion and Exclusion Criteria.

Inclusion Criteria	Exclusion Criteria
CBCT scans of oral and maxillofacial area for humans	Panoramic and CT scans of oral and maxillofacial area Inhumans
Diagnostic tool based on semi-automatic and fully automatic algorithm	CBCT scans of oral and maxillofacial area in animals
Experts judge or manual technique	Tracing any oral and maxillofacial structure rather than the IAN/IAC
Tracing the IAN/IAC	Pilot, ex-vivo studies, conference paper/review
Retrospective clinical trials, cross-sectional, case-control study	Full text not accessible
Studies published in any language and with the full text is accessible	
No date restriction	

**Table 2:** Data extracted from included studies. OMF, Oral and Maxillofacial.

Author, Study Location, and Year of Publication	Algorithm	Total Sample	Persons Executing and Interpreting Reference Tests		Software Used for Reference Test Method	Data Sets Used for Training, Validation and Test	Validation Technique	Sensitivity	Specificity	Accuracy	Agreement between Methods
			Number	Expertise							
Orhan et al., Turkey, 2021. [37]	U-net-like (Diagnocat ©)	85	1	OMF radiologist	N/A	N/A	N/A	N/A	N/A	N/A	Kappa statistics = 0.762
Liu et al., China, 2021. [38]	Two U-Net, One ResNet-34	229	2	OMF radiologists with 10 years of experience	Manually modification using Multi-Planar Reformation (MPR)	154, 30, 45 (train, valid, test)	Train, validation, and test split	90.2%	95.0%	93.3%	Kendall’s coefficient = 0.901
Bayrakdar et al., Turkey, 2021. [39]	U-net-like, (Diagnocat ©)	75	1	OMF radiologist with 8 years of experience	N/A	N/A	N/A	N/A	N/A	N/A	N/A
Kwak et al., Korea, 2020. [40]	2D SegNet, 2D U-Net, 3D U-Net	102	3	Two trained researchers, One OMF radiologist with 6 years of experience	INVIVO™(Anatomage, San Jose, CA, USA)	6:2:2 (train:valid:test)	Train, validation, and test split	N/A	N/A	96 % (2D SegNet), 84% (2D U-Net), 99% (3D U-Net)	N/A
Jaskari et al., Finland, 2020. [41]	Fully convolutional deep neural network	637	2	OMF radiologist with 34 years experience and resident in dental and maxillofacial radiologist with 10 years of experience	Planmeca Romexis^®^ 4.6.2.R software	457, 52, 128 (train, valid, test)	Train, validation, and test split	N/A	N/A	90%	N/A
Abdolali et al., Iran, 2016. [42]	Statistical shape models	120	2	Radiologists with at least 10 years of experience	N/A	84 (training set)	Leave-one-out cross-validation	N/A	N/A	N/A	N/A
Bahrampour et al., Iran, 2016 [43]	Automated algorithm	40	2	Maxillofacial radiologists	N/A	N/A	N/A	N/A	N/A	N/A	N/A

## Data Availability

The data that support the findings of this study are available from the corresponding author upon reasonable request.

## Supplementary Materials

The following are available online at https://www.mdpi.com/article/10.3390/ijerph19010560/s1, Table S1: Searching stagey; Table S2: QUADAS-2 tool questions.

## References

[1] Amisha Malik P., Pathania M., Rathaur V.K. (2019). Overview of artificial intelligence in medicine. J. Fam. Med. Prim. Care.

[2] Panch T., Szolovits P., Atun R. (2018). Artificial intelligence, machine learning and health systems. J. Glob. Health.

[3] Helm J.M., Swiergosz A.M., Haeberle H.S., Karnutaet J.M., Schaffer J.L., Krebs V.E., Spitzer A.I., Ramkumar P.N. (2020). Machine Learning and Artificial Intelligence: Definitions, Applications, and Future Directions. Curr. Rev. Musculoskelet. Med..

[4] Lee R.S.T. (2020). Artificial Intelligence in Daily Life.

[5] Lee D., Yoon S.N. (2021). Application of Artificial Intelligence-Based Technologies in the Healthcare Industry: Opportunities and Challenges. Int. J. Environ. Res. Public Health.

[6] Bohr A., Memarzadeh K. (2020). The rise of artificial intelligence in healthcare applications. Artificial Intelligence in Healthcare.

[7] Benke K., Benke G. (2018). Artificial Intelligence and Big Data in Public Health. Int. J. Environ. Res. Public Health.

[8] Hashimoto D.A., Rosman G., Rus D., Meireles O.R. (2018). Artificial Intelligence in Surgery: Promises and Perils. Ann. Surg..

[9] Mintz Y., Brodie R. (2019). Introduction to artificial intelligence in medicine. Minim. Invasive Ther. Allied Technol..

[10] Ramesh A.N., Kambhampati C., Monson J.R., Drew P.J. (2004). Artificial intelligence in medicine. Ann. R. Coll. Surg. Engl..

[11] Hassani H., Andi P.A., Ghodsi A., Norouzi K., Komendantova N., Unger S. (2021). Shaping the Future of Smart Dentistry: From Artificial Intelligence (AI) to Intelligence Augmentation (IA). IoT.

[12] Samiuddin Ahmed M., Chaturya K., Vinay Chandra Tiwari R., Virk I., Kumar Gulia S., Rajkumar Pandey P., Tiwari H. (2020). Digital Dentistry-New Era in Dentistry. J. Adv. Med. Dent. Sci. Res..

[13] Krishna A.B., Tanveer A., Bhagirath P.V., Gannepalli A. (2020). Role of artificial intelligence in diagnostic oral pathology—A modern approach. J. Oral Maxillofac. Pathol..

[14] Kar A., Wreesmann V.B., Shwetha V., Thakur S., Rao V.U., Arakeri G., Brennan P.A. (2020). Improvement of oral cancer screening quality and reach: The promise of artificial intelligence. J. Oral Pathol. Med..

[15] Alkilzy M., Midani R., Höfer M., Splieth C. (2019). Improving Toothbrushing with a Smartphone App: Results of a Randomized Controlled Trial. Caries Res..

[16] Klingberg G., Sillén R., Norén J.G. (1999). Machine learning methods applied on dental fear and behavior management problems in children. Acta Odontol. Scand..

[17] Aminoshariae A., Kulild J., Nagendrababu V. (2021). Artificial Intelligence in Endodontics: Current Applications and Future Directions. J. Endod..

[18] Putra R.H., Doi C., Yoda N., Astuti E.R., Sasaki K. (2021). Current applications and development of artificial intelligence for digital dental radiography. Dentomaxillofac. Radiol..

[19] Brown J., Jacobs R., Levring Jäghagen E., Lindh C., Baksi G., Schulze D., Schulze R., European Academy of DentoMaxilloFacial Radiology (2014). Basic training requirements for the use of dental CBCT by dentists: A position paper prepared by the European Academy of DentoMaxilloFacial Radiology. Dentomaxillofac. Radiol..

[20] Macleod I., Heath N. (2008). Cone-beam computed tomography (CBCT) in dental practice. Dent Update.

[21] Hung K., Yeung A.W.K., Tanaka R., Bornstein M.M. (2020). Current Applications, Opportunities, and Limitations of AI for 3D Imaging in Dental Research and Practice. Int. J. Environ. Res. Public Health.

[22] Nagi R., Aravinda K., Rakesh N., Gupta R., Pal A., Mann A.K. (2020). Clinical applications and performance of intelligent systems in dental and maxillofacial radiology: A review. Imaging Sci. Dent.

[23] Nguyen J.D., Duong H. Anatomy, Head and Neck, Alveolar Nerve. StatPearls. https://www.ncbi.nlm.nih.gov/books/NBK546712/.

[24] Wolf K.T., Brokaw E.J., Bell A., Joy A. (2016). Variant Inferior Alveolar Nerves and Implications for Local Anesthesia. Anesth. Prog..

[25] Ozturk A., Potluri A., Vieira A.R. (2012). Position and course of the mandibular canal in skulls. Oral Surg. Oral Med. Oral Pathol. Oral Radiol..

[26] Shavit I., Juodzbalys G. (2014). Inferior alveolar nerve injuries following implant placement—Importance of early diagnosis and treatment: A systematic review. J. Oral Maxillofac. Res..

[27] Rood J.P., Shehab B.A. (1990). The radiological prediction of inferior alveolar nerve injury during third molar surgery. Br. J. Oral Maxillofac. Surg..

[28] Kaasalainen T., Ekholm M., Siiskonen T., Kortesniemi M. (2021). Dental cone beam CT: An updated review. Phys. Med..

[29] Weckx A., Agbaje J.O., Sun Y., Jacobs R., Politis C. (2016). Visualization techniques of the inferior alveolar nerve (IAN): A narrative review. Surg. Radiol. Anat..

[30] Hung K., Montalvao C., Tanaka R., Kawai T., Bornstein M.M. (2020). The use and performance of artificial intelligence applications in dental and maxillofacial radiology: A systematic review. Dentomaxillofac. Radiol..

[31] Fletcher R.H., Fletcher S.W., Fletcher G.S. (2012). Clinical Epidemiology: The Essentials. Diagnosis.

[32] Campbell J.M., Klugar M., Ding S., Carmody D.P., Hakonsen S.J., Jadotte Y.T., White S., Munn Z. (2015). Diagnostic test accuracy: Methods for systematic review and meta-analysis. Int. J. Evid. Based Healthc..

[33] Page M.J., McKenzie J.E., Bossuyt P.M., Boutron I., Hoffmann T.C., Mulrow C.D. (2021). The PRISMA 2020 statement: An updated guideline for reporting systematic reviews. BMJ.

[34] Munn Z., Stern C., Aromataris E., Lockwood C., Jordan Z. (2018). What kind of systematic review should I conduct? A proposed typology and guidance for systematic reviewers in the medical and health sciences. BMC Med. Res. Methodol..

[35] Bramer W.M., Giustini D., de Jonge G.B., Holland L., Bekhuis T. (2016). De-duplication of database search results for systematic reviews in EndNote. J. Med. Libr. Assoc..

[36] Ma L.L., Wang Y.Y., Yang Z.H., Huang D., Weng H., Zeng X.T. (2020). Methodological quality (risk of bias) assessment tools for primary and secondary medical studies: What are they and which is better?. Mil. Med. Res..

[37] Orhan K., Bilgir E., Bayrakdar I.S., Ezhov M., Gusarev M., Shumilov E. (2021). Evaluation of artificial intelligence for detecting impacted third molars on cone-beam computed tomography scans. J. Stomatol. Oral Maxillofac. Surg..

[38] Liu M.Q., Xu Z.N., Mao W.Y., Li Y., Zhang X.H., Bai H.L., Ding P., Fu K.Y. (2021). Deep learning-based evaluation of the relationship between mandibular third molar and mandibular canal on CBCT. Clin. Oral Investig..

[39] Bayrakdar S.K., Orhan K., Bayrakdar I.S., Bilgir E., Ezhov M., Gusarev M., Shumilov E. (2021). A deep learning approach for dental implant planning in cone-beam computed tomography images. BMC Med. Imaging.

[40] Kwak G.H., Kwak E.J., Song J.M., Park H.R., Jung Y.H., Cho B.H., Hui P., Hwang J.J. (2020). Automatic mandibular canal detection using a deep convolutional neural network. Sci. Rep..

[41] Jaskari J., Sahlsten J., Järnstedt J., Mehtonen H., Karhu K., Sundqvist O., Hietanen A., Varjonen V., Mattila V., Kaski K. (2020). Deep Learning Method for Mandibular Canal Segmentation in Dental Cone Beam Computed Tomography Volumes. Sci. Rep..

[42] Abdolali F., Zoroofi R.A., Abdolali M., Yokota F., Otake Y., Sato Y. (2017). Automatic segmentation of mandibular canal in cone beam CT images using conditional statistical shape model and fast marching. Int. J. Comput. Assist. Radiol. Surg..

[43] Bahrampour E., Zamani A., Kashkouli S., Soltanimehr E., Jahromi M.G., Pourshirazi Z.S. (2016). Accuracy of software designed for automated localization of the inferior alveolar nerve canal on cone beam CT images. Dento Maxillo Facial Radiol..

[44] Cohen J.F., Korevaar D.A., Altman D.G., Bruns D.E., Gatsonis C.A., Hooft L., Irwig L., Levine D., Reitsma J.B., Bossuyt P.M. (2016). STARD 2015 guidelines for reporting diagnostic accuracy studies: Explanation and elaboration. BMJ Open.

[45] Ying X. (2019). An Overview of Overfitting and its Solutions. J. Phys. Conf. Ser..

[46] McHugh M.L. (2012). Interrater reliability: The kappa statistic. Biochem. Med..

[47] Innes E., Straker L. (1999). Reliability of work-related assessments. Work.

[48] Eusebi P. (2013). E-Mail Methodological Notes Diagnostic Accuracy Measures. Cerebrovasc. Dis..

[49] Schulze R., Heil U., Groβ D., Bruellmann D.D., Dranischnikow E., Schwanecke U., Schoemer E. (2011). Artefacts in CBCT: A review. Dentomaxillofacial Radiol..

[50] Sounderajah V., Ashrafian H., Rose S., Shah N.H., Ghassemi M., Golub R., Kahn C.E., Esteva A., Karthikesalingam A., Mateen B. (2021). A quality assessment tool for artificial intelligence-centered diagnostic test accuracy studies: QUADAS-AI. Nat. Med..

[51] Sounderajah V., Ashrafian H., Aggarwal R., de Fauw J., Denniston A.K., Greaves F., Karthikesalingam A., King D., Liu X., Markar S.R. (2020). Developing specific reporting guidelines for diagnostic accuracy studies assessing AI interventions: The STARD-AI Steering Group. Nat. Med..

[52] Rivera S.C., Liu X., Chan A.W., Denniston A.K., Calvert M.J. (2020). Guidelines for clinical trial protocols for interventions involving artificial intelligence: The SPIRIT-AI extension. Nat. Med..

[53] Liu X., Rivera S.C., Moher D., Calvert M.J., Denniston A.K. (2020). Reporting guidelines for clinical trial reports for interventions involving artificial intelligence: The CONSORT-AI extension. Nat. Med..

[54] Clinical-Trials.ai|Home n.d. https://www.clinical-trials.ai/.

[55] Schwendicke F., Singh T., Lee J.H., Gaudin R., Chaurasia A., Wiegand T., Uribe S., Krois J. (2021). Artificial intelligence in dental research: Checklist for authors, reviewers, readers. J. Dent..

