# Supplementary material for: The Effectiveness of Semi-Automated and Fully Automatic Segmentation for Inferior Alveolar Canal Localization on CBCT Scans: A Systematic Review
Source: Int J Environ Res Public Health. 2022 Jan 4;19(1):560. doi: 10.3390/ijerph19010560 (PMC8744855; doi:10.3390/ijerph19010560)
Supplement: Supplementary file 1 [file ijerph-19-00560-s001.zip › Table S1.pdf]

Searching stagey:

Searched database: PubMed, Medline, Web of Science, Cochrane (Wiley) and Scopus
Searching Date: 22-08-2021
No filters applied and no publication date restrictions.

1- PubMed:

Search	Query	Field	Records retrieved
#1	algorithm OR algorithm* OR "artificial intelligence" OR AI OR automatic OR automated OR semi-automatic OR semi-automated OR "deep learning" OR "Convolutional neural network" OR CNN OR "machine learning"	All Field	1,761,241
#2	"mandibular canal" OR "inferior alveolar canal" OR "inferior alveolar nerve	All Field	3,872
#3	#1 AND #2<br>(algorithm OR algorithm* OR "artificial intelligence" OR AI OR automatic OR automated OR semi-automatic OR semi-automated OR "deep learning" OR "Convolutional neural network" OR CNN OR "machine learning") AND ("mandibular canal" OR "inferior alveolar canal" OR "inferior alveolar nerve")		78

2- Medline (Ovid):

Search	Query	Field	Records retrieved
#1	algorithm OR algorithm* OR artificial intelligence OR AI OR automatic OR automated OR semi-automatic OR semi-automated OR deep learning OR Convolutional neural network OR CNN OR machine learning	.mp. [mp=title, abstract, original title, name of substance word, subject heading word, floating sub-	553,434

		heading word, keyword heading word, organism suppleme ntary concept word, protocol suppleme ntary concept word, rare disease suppleme ntary concept word, unique identifier, synonyms]	
#2	mandibular canal or inferior alveolar canal or inferior alveolar nerve	.mp. [mp=title, abstract, original title, name of substance word, subject heading word, floating sub-heading word, keyword heading word, organism suppleme	3,281

		ntary concept word, protocol suppleme ntary concept word, rare disease suppleme ntary concept word, unique identifier, synonyms]	
#3	#1 AND #2		46

Cochrane (Wiley):

Search	Query	Field	Records retrieved
#1	algorithm OR algorithm* OR “artificial intelligence” OR AI OR automatic OR automated OR semi-automatic OR semi-automated OR deep learning OR “Convolutional neural network” OR CNN OR “machine learning”	Title/Abstract/Key words	36,696
#2	“mandibular canal” or “inferior alveolar canal” or “inferior alveolar nerve”	Title/Abstract/Key words	757
#3	#1 AND #2		3

Web of Science:

Search	Query	Field	Records retrieved
#1	ALL=(algorithm OR algorithm* OR “artificial intelligence” OR AI OR automatic OR automated OR semi-automatic OR semi-automated OR “deep learning” OR “Convolutional neural network” OR CNN OR “machine learning”)	Indexes=SCI-EXPANDED,	3,698,137

		SSCI, A&HCI, CPCI-S, CPCI-SSH, BKCI-S, BKCI-SSH, ESCI, CCR-EXPANDED, IC Timespan=All	
#2	ALL=("mandibular canal" OR "inferior alveolar canal" OR "inferior alveolar nerve")	Indexes=SCI-EXPANDED, SSCI, A&HCI, CPCI-S, CPCI-SSH, BKCI-S, BKCI-SSH, ESCI, CCR-EXPANDED, IC Timespan=All	3,717
#3	#1 AND #2<br>(ALL=(algorithm OR algorithm* OR "artificial intelligence" OR AI automatic OR automated OR semi-automatic OR semi-automated		50

	OR “deep learning” OR “Convolutional neural network” OR CNN OR “machine learning”)) AND ALL=(“mandibular canal” OR “inferior alveolar canal” OR “inferior alveolar nerve”)	

Scopus: 19/08/2021

Search	Query	Field	Records retrieved
#1	ALL (algorithm OR algorithm* OR "artificial intelligence" OR ai OR automatic OR automated OR semi-automatic OR semi-automated OR "deep learning" OR "Convolutional neural network" OR cnn OR “machine learning”)	All Field	10,303,872
#2	ALL ("mandibular canal" OR "inferior alveolar canal" OR "inferior alveolar nerve")	All Field	4,820
#3	#1 AND #2<br>(ALL (algorithm OR algorithm* OR "artificial intelligence" OR ai OR automatic OR automated OR semi-automatic OR semi-automated OR "deep learning" OR "Convolutional neural network" OR cnn OR “machine learning”) AND ALL ("mandibular canal" OR "inferior alveolar canal" OR "inferior alveolar nerve"))		813
