# Supplementary material for: The Effectiveness of Semi-Automated and Fully Automatic Segmentation for Inferior Alveolar Canal Localization on CBCT Scans: A Systematic Review
Source: Int J Environ Res Public Health. 2022 Jan 4;19(1):560. doi: 10.3390/ijerph19010560 (PMC8744855; doi:10.3390/ijerph19010560)
Supplement: Supplementary file 1 [file ijerph-19-00560-s001.zip › Table S2.pdf]

QUADAS-2 tool questions

		Yes	No	Unclear	Not applicable
Patient selection	Was a consecutive or random sample of patients enrolled?				
	Was a case-control design avoided?				
	Did the study avoid inappropriate exclusions?				
Index tests	Were the index test results interpreted without knowledge of the results of the reference standard?				
	If a threshold was used, was it pre-specified?				
Reference standard/test	Is the reference standard likely to correctly classify the target condition?				
	Were the reference standard results interpreted without knowledge of the results of the index test?				
Flow and timing	Was there an appropriate interval between the index test and reference standard?				
	Did all patients receive the same reference standard?				
	Were all patients included in the analysis?				
